# Eating Difficulties among Older Adults with Dementia in South Korean Long-Term Care Facilities: A Scoping Review

**DOI:** 10.1093/geroni/igab046.2458

**Published:** 2021-12-17

**Authors:** Eunju Choi, Dukyoo Jung, Kyuri Lee

**Affiliations:** 1 College of Nursing, Ewha Womans University, Seoul, Seoul-t'ukpyolsi, Republic of Korea; 2 Ewha Womans University, Seoul, Seoul-t'ukpyolsi, Republic of Korea

## Abstract

This study aims to synthesize existing literature concerning eating difficulties among older adults with dementia in long-term care facilities. A scoping review, using the framework proposed by Arksey & O'Malley (2005) and improved and supplemented by Levac et al. (2010), was conducted. Literature was searched from five bibliographic databases—Research Information Sharing Service (RISS), Korean studies Information Service System (KISS), National Digital Science Library (NDSJ), Korean Medical Database (KMBASE), DataBase Periodical Information Academic(DBPia), Google Scholar, and gray literature. Literature selection and characteristics were approved by two independent reviewers, using pre-tested forms to determine final inclusion. Eventually, 111 articles from 2012–2020 were identified, and the 11 articles were used for the final analysis. We found that primarily utilized Eating behavior scale (EBS) and Edinburgh feeding evaluation in dementia scale (EdFED) had utilized as measurement tools for evaluating eating behavior. The most common factors related to eating behavior of older adults with dementia included cognitive and physical functions in the individual domain, the caregiver's attitude toward eating in the inter-individual domain, and types of meal in the environmental domain. Therefore, it is essential to develop measurement tools that reflect the eating behavior of older adults with dementia, a comprehensive understanding of the eating behavior of old adults with dementia, and create effective interventions that can be implemented in the specificity of long-term care facilities in Korea. The results of this analysis are intended to be used as basis to develop a meal support programs for older adults with dementia.

